# Periodontal inflamed surface area is associated with hs-CRP in septuagenarian Japanese adults in cross-sectional findings from the SONIC study

**DOI:** 10.1038/s41598-021-93872-8

**Published:** 2021-07-14

**Authors:** Koji Miki, Masahiro Kitamura, Kodai Hatta, Kei Kamide, Yasuyuki Gondo, Motozo Yamashita, Masahide Takedachi, Takenori Nozaki, Chiharu Fujihara, Yoichiro Kashiwagi, Tomoaki Iwayama, Toshihito Takahashi, Hitomi Sato, Yuki Murotani, Mai Kabayama, Yasushi Takeya, Yoichi Takami, Hiroshi Akasaka, Koichi Yamamoto, Ken Sugimoto, Tatsuro Ishizaki, Yukie Masui, Hiromi Rakugi, Kazunori Ikebe, Shinya Murakami

**Affiliations:** 1 Department of Periodontology, Osaka University Graduate School of Dentistry, 1-8, Yamadaoka, Suita, Osaka 565-0871 Japan; 2 Department of Prosthodontics, Gerodontology and Oral Rehabilitation, Osaka University Graduate School of Dentistry, Suita, Osaka Japan; 3 Division of Health Science, Osaka University Graduate School of Medicine, Suita, Osaka Japan; 4 Department of Clinical Thanatology and Geriatric Behavioral Science, Osaka University Graduate School of Human Science, Suita, Osaka Japan; 5 Division for Interdisciplinary Dentistry, Osaka University Dental Hospital, Suita, Osaka Japan; 6 Department of Geriatric and General Medicine, Osaka University Graduate School of Medicine, Suita, Osaka Japan; 7 Department of General and Geriatric Medicine, Kawasaki Medical University, Okayama, Okayama Japan; 8 Tokyo Metropolitan Geriatric Hospital and Institute of Gerontology, Itabashi-ku, Tokyo Japan

**Keywords:** Biomarkers, Diseases, Health care, Medical research

## Abstract

Periodontal disease is a chronic inflammatory condition that affects various peripheral organs. The periodontal inflamed surface area (PISA) quantifies periodontitis severity and the spread of inflammatory wounds. This study aimed to investigate the association between PISA and high-sensitivity C-reactive protein (hs-CRP), a systemic inflammation marker. This study included 250 community-dwelling septuagenarians (69–71 years). We collected information on their medical (e.g., diabetes and dyslipidemia) and dental examinations (e.g., measurement of the probing pocket depth). Generalized linear model analysis was used to explore the association between PISA and hs-CRP levels. There was a significant difference in hs-CRP levels between groups with PISA ≥ 500 and < 500 (p = 0.017). Moreover, the generalized linear model analysis revealed a significant association between PISA and hs-CRP levels (risk ratio = 1.77; p = 0.033) even after adjusting other factors. Further, we found a correlation between PISA and hs-CRP (Spearman’s rank correlation coefficient, rs = 0.181; p = 0.023). Our findings suggest that PISA is an effective index for estimating the effect of periodontitis on the whole body, enabling medical-dental cooperation.

## Introduction

Periodontal disease is a chronic inflammatory disease caused by a periodontopathic bacterial infection, which causes periodontal tissue destruction. Periodontal pockets are formed in periodontitis-affected tissues; moreover, there is proliferation of subgingival plaques. An ulcer surface is formed on the inner surface of the periodontal pocket by disrupting the continuity of epithelial cells, which results in easy bleeding. This indicates that a mild chronic inflammatory condition persists throughout the body, not just in the periodontal tissue. Moreover, periodontitis increases the risk of the development and progression of various diseases, including type 2 diabetes, arteriosclerosis, rheumatoid arthritis, and chronic kidney disease^1–7^.

Compared with that of healthy individuals, patients with severe periodontitis have been shown to present significantly increased serum C-reactive protein (CRP) levels^8–13^. However, the gingival inflammation index, an inflammation index of conventional periodontal tissue, and bleeding on probing (BOP) are unfamiliar to most medical personnel and are not quantitatively evaluated. Therefore, an objective index could provide information accessible to the medical department.

The periodontal inflamed surface area (PISA) reflects the surface area of the bleeding pocket epithelium in square millimeters. It is measured using conventional clinical parameters of periodontal tissue status, specifically, BOP combined with either periodontal pocket depth (PPD) or clinical attachment loss and gingival recession^14^.

The PISA can quantify the periodontitis severity and extent of inflammatory wounds. There are currently various clinical studies on the association between PISA and risk of systemic diseases^15–17^. The PISA has been reported to be associated with high-sensitivity CRP (hs-CRP), which is a systemic inflammatory marker^18,19^.

However, these studies targets patients with systemic diseases such as diabetes and kidney disease, and there are no studies investigating the relationship between PISA and hs-CRP as a health survey in general local residents.

The standard value of PISA that raises hs-CRP is not clear; however, it has been reported that it may significantly raised around PISA = 500 mm^2^^18^. Few studies have investigated on the relationship between PISA and hs-CRP, a marker of systemic inflammation, and further studies are needed.

This study, targeting 70-year-old Japanese local residents, aimed to minimize the variation in the effects of age on CRP and investigate the association between PISA and hs-CRP.

## Materials and methods

### Study design and population

The current study is part of a prospective study investigating health and longevity called the Septuagenarians, Octogenarians, Nonagenarians Investigation with Centenarians (SONIC) Study. We randomly recruited 250 community-dwelling septuagenarians (69–71 years) from the Basic Resident Registry of Itami City in Hyogo Prefecture, which is a western urban area of Japan. Data were collected in 2010 at a local community hall.

The Institutional Review Board of Osaka University Graduate Schools of Dentistry approved all study protocols (approval number H22-E9), which were conducted in accordance with the principles of the Declaration of Helsinki. Furthermore, this study followed the Strengthening the Reporting of Observational Studies in Epidemiology (STROBE) guidelines^20^. Written informed consent was obtained from all participants prior to this study.

### Dental examinations

The periodontal condition was assessed by measuring PPD using a periodontal probe (CP‐12; Hu‐Friedy Mfg. Co., LLC). PPD was assessed at six sites (mesio-buccal, mid-buccal, disto-buccal, mesio-lingual, mid-lingual, and disto-lingual) for the remaining teeth. Regarding PPD measurement and BOP-positive site measurement, dentists who were trained in optimizing probing pressure from the Department of Periodontology, Osaka University Graduate School of Dentistry, performed a periodontal examination. On the day of the inspection, probing was calibrated by the measurer to a probing pressure of 25 g. The mean PPD, BOP, and the number of teeth with PPD ≥ 4 mm or PPD ≥ 6 mm were used as indicators of periodontal status. Additionally, the PISA was measured for all participants. It was calculated using an automatically computable EXCEL form known as the Calculate PISA Probing Pocket Depth as an alternative to the parsprototo.info website.

### Systemic factors

Diabetes mellitus was defined as fasting plasma glucose level ≥ 126 mg/dL and/or HbA1c level ≥ 6.1% and/or current medications for diabetes mellitus^21^. Dyslipidemia was defined as levels of low-density lipoprotein cholesterol ≥ 140 mg/dL, triglycerides ≥ 150 mg/dL, high-density lipoprotein cholesterol < 40 mg/dL, and/or medications for dyslipidemia^22,23^.

### Social and medical interviews

During the interviews, social and medical characteristics were assessed as well. The participants’ education level was categorized based on the duration: < 10 years, 10–12 years, and > 12 years. The history of smoking and malignant tumors was classified as yes or no.

### Statistical analysis

Between-group (PISA ≥ 500 and PISA < 500 groups) comparisons for continuous and categorical variables were performed using Mann–Whitney U-tests and chi-square tests, respectively. Generalized linear models with log link and gamma distribution were conducted to explore the association between PISA and hs-CRP. Risk ratio, corresponding 95% confidence intervals (CIs), and P-values for each covariate were reported. Spearman’s rank correlation coefficients were used to investigate the association between the conventional periodontal disease test and PISA.

All statistical analyses were performed using SPSS Statistics 25 (IBM Japan). Statistical significance was set at P < 0.05.

## Results

**Figure 1:**
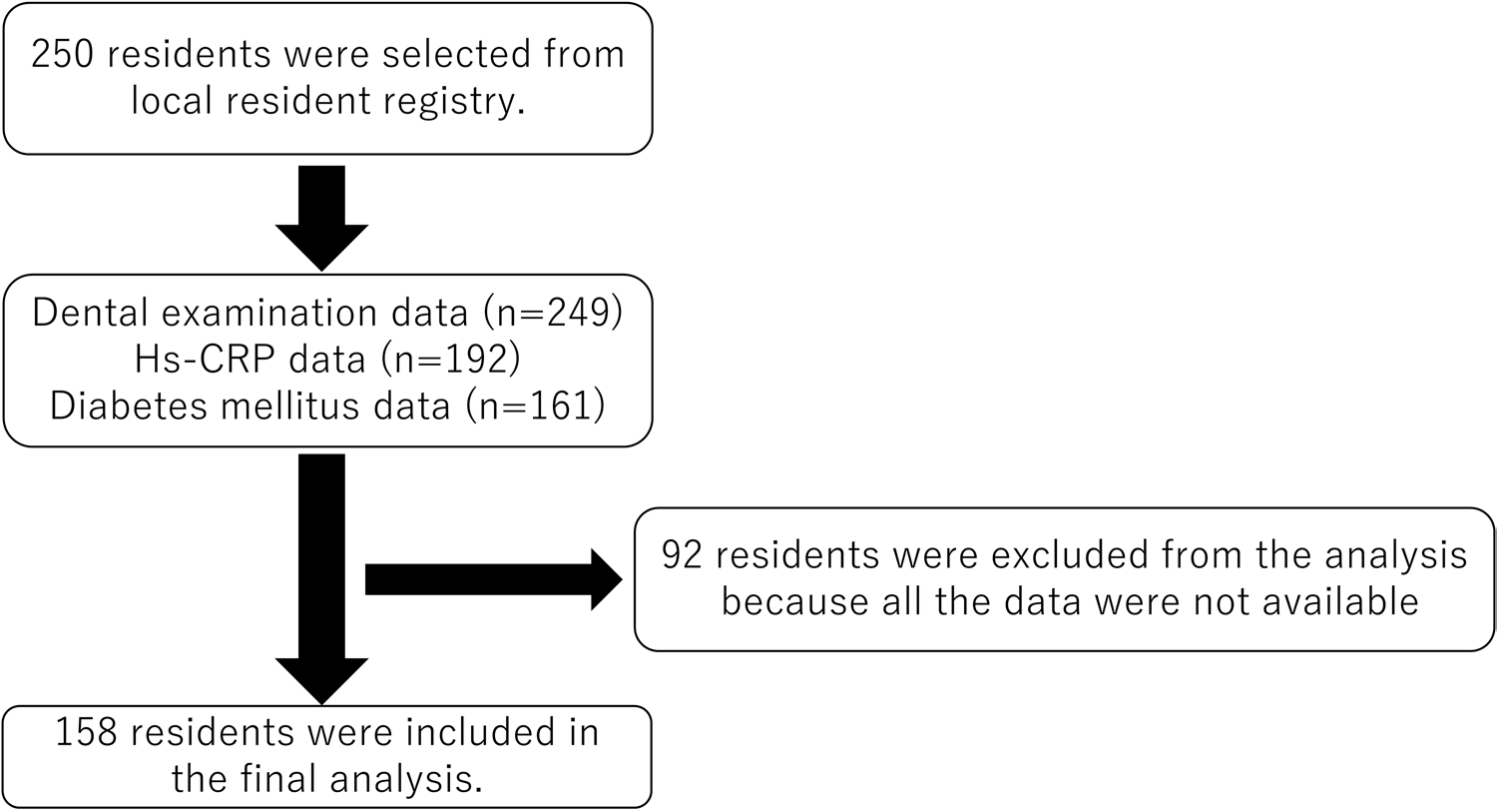
Overview of the study design. We randomly recruited 250 residents (69–71 years) from the Basic Resident Registry of Itami City in Japan. Among them, 249 participants had undergone dental examinations. Data regarding hs-CRP and presence/absence of diabetes mellitus were obtained from 192 and 161 participants, respectively. We were able to obtain data for all items in 158 participants, who were included in the final analysis.

**Figure 2:**
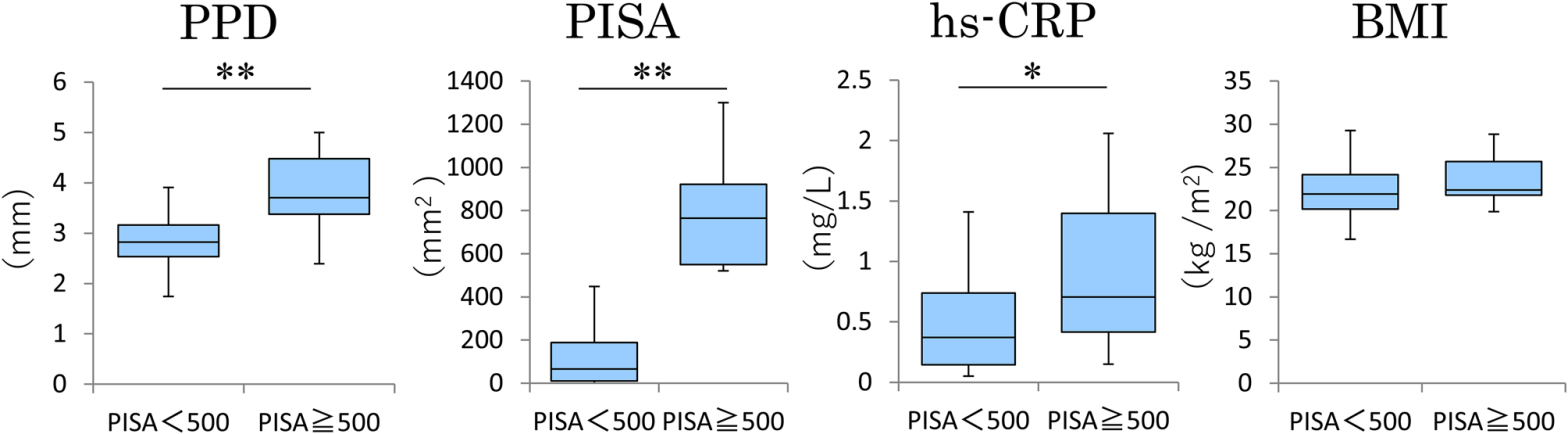
Box plots of mean PPD, PISA, hs-CRP level, and BMI in the PISA < 500 and PISA ≥ 500 groups. *p < 0.05, **p < 0.01 (Mann–Whitney U-test).

Figure 1 presents the sample flowchart. A total of 249 of the 250 participants underwent dental examination. Data regarding hs-CRP levels and diabetes status were obtained from 192 and 161 participants, respectively. All data were obtained from 158 participants. Table 1 shows the clinical profiles of the participants. There were no significant between-group differences in sex, years of education, smoking, body mass index (BMI), diabetes mellitus, malignancy, or dyslipidemia. The median hs-CRP levels in the PISA < 500 and PISA ≥ 500 groups were 0.37 mg/L and 0.71 mg/L, respectively. Figure 2 shows box plots of the mean PPD, PISA, hs-CRP level, and BMI in both groups. The median BMI in the PISA < 500 and PISA ≥ 500 groups was 21.9 kg/m^2^ and 22.4 kg/m^2^, respectively. A significant between-group difference was noted in the hs-CRP levels (p = 0.017, Mann–Whitney U-test). Table 2 presents the generalized linear model analysis with the hs-CRP level as the objective variable, PISA as the explanatory variable, and basic attributes and other possible inflammation-related diseases as adjustment variables to determine whether periodontal disease explains the systemic inflammatory state. The PISA was significantly associated with hs-CRP levels (risk ratio, RR = 1.77; p = 0.033). Table 3 shows the correlation coefficients among the number of teeth with PPD ≥ 6 mm or PPD ≥ 4 mm, PISA, and hs-CRP levels. Hs-CRP level was significantly associated with PISA (Spearman’s rank correlation coefficient, rs = 0.181; p = 0.023) but not the number of teeth with PPD ≥ 6 mm.

**Table 1:** Comparison of the characteristics between PISA < 500 group and PISA ≥ 500 group (n = 158).

Characteristic	N (%)	PISA < 500 n = 142	PISA≧500 n = 16	*P-*value
**Sex, n (%)**				
Male	81 (51.3)	76 (53.5)	5 (31.3)	0.091^†^
Female	77 (48.7)	66 (46.5)	11 (68.8)	
**Education year, n (%)**				
≦ 9 years	31 (19.6)	27 (19.0)	4 (25.0)	0.607^†^
10–12 years	70 (44.3)	62 (43.7)	8 (50.0)	
≧ 13 years	57 (36.1)	53 (37.3)	4 (25.0)	
**Smoking habit, n (%)**				
No	144 (91.1)	129 (90.8)	15 (93.8)	0.698^†^
Yes	14 (8.9)	13(9.2)	1 (6.3)	
**BMI, n (%)**				
< 25 kg/m^2^	132 (83.5)	120 (84.5)	12 (75.0)	0.331^†^
≧ 25 kg/m^2^	26 (16.5)	22 (15.5)	4 (25.0)	
**Diabetes mellitus, n (%)**				
No	131 (82.9)	117 (82.4)	14 (87.5)	0.607^†^
Yes	27 (17.1)	25 (17.6)	2 (12.5)	
**Malignant tumor, n (%)**				
No	143 (90.5)	129 (90.8)	14 (87.5)	0.665^†^
Yes	15 (9.5)	13 (9.2)	2 (12.5)	
**Dyslipidemia, n (%)**				
No	70 (44.3)	64 (45.1)	6 (37.5)	0.563^†^
Yes	88 (55.7)	78 (54.9)	10 (62.5)	

		Median (IQR)	Median (IQR)	
Mean PPD (mm)		2.8 (2.4–3.1)	3.7 (3.4–4.5)	< 0.001^§^
Remaining teeth		25.0 (17.0–27.0)	27.0 (21.8–28.0)	0.179^§^
PISA (mm^2^)		65.6 (10.0–192.0)	765.1 (548.3–928.7)	< 0.001^§^
hs-CRP (mg/L)		0.37 (0.14–0.74)	0.71 (0.37–1.59)	0.017^§^

*PISA* periodontal inflamed surface area, *BMI* body mass index, *IQR* interquartile range, *PPD* probing pocket depth, *hs-CRP* high-sensitivity C-reactive protein.

*p* values determined using ^†^ chi-square tests for categorical variables and ^§^ Mann–Whitney U test for continuous variables.

**Table 2:** Results of the generalized linear models with log link and gamma distribution for each variables on hs-CRP.

Variable	hs-CRP		hs-CRP		hs-CRP		hs-CRP	
	RR (95% CI)	*p*-value	RR (95% CI)	*p*-value	RR (95% CI)	*p*-value	RR (95% CI)	*p*-value
Sex (reference: men)	0.72 (0.51–1.02)	0.062	0.73 (0.52–1.03)	0.069	0.76 (0.54–1.08)	0.129	0.77 (0.54–1.09)	0.138
Education years (reference: ≤ 9 years) 10–12 years	0.83 (0.53–1.30)	0.414	0.82 (0.52–1.28)	0.382	0.84 (0.54–1.33)	0.464	0.84 (0.53–1.33)	0.453
≥ 13 years	0.70 (0.44–1.11)	0.128	0.69 (0.43–1.09)	0.110	0.71 (0.44–1.13)	0.150	0.70 (0.44–1.12)	0.140
Smoking habit (reference: no)	1.29 (0.71–2.38)	0.405	1.26 (0.68–2.32)	0.458	1.25 (0.67–2.31)	0.480	1.28 (0.69–2.37)	0.440
Diabetes (reference: no)	0.87 (0.56–1.35)	0.536	0.87 (0.57–1.34)	0.539	0.83 (0.54–1.29)	0.410	0.85 (0.55–1.32)	0.463
Dyslipidemia (reference: no)	0.84 (0.61–1.14)	0.257	0.80 (0.59–1.10)	0.172	0.84 (0.61–1.16)	0.288	0.86 (0.63–1.18)	0.353
History of cancer (reference: no)	0.92 (0.54–1.58)	0.771	0.79 (0.45–1.36)	0.388	0.96 (0.55–1.68)	0.893	1.01 (0.60–1.73)	0.957
BMI (reference: < 25 kg/m^2^)	1.39 (0.92–2.10)	0.122	1.36 (0.90–2.05)	0.147	1.48 (0.97–2.24)	0.068	1.50 (0.99–2.28)	0.055
PISA (reference: < 500)	1.77 (1.05–2.98)	0.033						
The number of BOP positive site			1.01 (1.00–1.02)	0.012				
The number of PPD ≥ 4 mm teeth					1.01 (0.99–1.03)	0.246		
The number of PPD ≥ 6 mm teeth							1.03 (0.98–1.08)	0.238

*Hs-CRP* high-sensitivity C-reactive protein, *RR* risk ratio, *CI* confidence interval, *BMI* body mass index, *BOP* bleeding on probing.

**Table 3:** Spearman’s rank correlation coefficients between severity of periodontitis and hs-CRP (n = 158).

Variables	1	2	3	4
1. PISA				
2. The number of PPD ≥ 4 mm teeth	0.719**			
3. The number of PPD ≥ 6 mm teeth	0.692**	0.670**		
4. hs-CRP	0.181*	0.164*	0.152	

*PISA* periodontal inflamed surface area, *PPD* probing pocket depth, *hs-CRP* high-sensitivity C-reactive protein.

*p < 0.05; **p < 0.01.

## Discussion

This study found a significant association between PISA and hs-CRP in community-dwelling septuagenarians. Previous studies have reported an association between PISA and CRP^18,19^, however, to our knowledge, this is the only age-fixed related study. CRP levels are positively correlated with age^24,25^. This study minimized the effect of age variability on hs-CRP, which allowed more accurate analysis of the effects of periodontitis on the whole body.

We believe that this study yielded higher evidence because it is based on a real population of 70-year-olds living in the community, rather than an age-adjusted multiple regression analysis that showed a significant association with CRP. CRP can be a useful marker for systemic inflammation as well as the risk of coronary artery disease^26,27^. Compared with that of healthy individuals, patients with periodontal disease present with significantly increased serum CRP levels^28^. Moreover, numerous studies have reported a positive relationship of periodontal disease morbidity with inflammatory cytokines, including tumor necrosis factor, interleukin (IL)-1, IL-6, and IL-8, which are involved in arteriosclerosis. However, no consensus has been reached^10,29,30^. There is a need for further studies to determine whether the increase in inflammatory markers caused by periodontal disease is quantitatively sufficient to cause atherosclerosis onset and progression. Given that the arteriosclerosis causes cannot be explained by classical risk factors, including hypertension, dyslipidemia, and diabetes, arteriosclerosis could be associated with periodontal disease^31,32^. Additionally, periodontal disease treatments reduce the levels of IL-6, CRP, etc.^33–36^.

In this study, the mean and median hs-CRP levels in the PISA < 500 group were 0.609 ± 0.763 mg/L and 0.37 mg/L, respectively, while the corresponding values in the PISA ≥ 500 group were 0.958 ± 0.792 mg/L and 0.71 mg/L, respectively. Compared with that of the PISA < 500 group, the mean hs-CRP level of the PISA ≥ 500 group was higher by approximately 0.35 mg/L. The hs-CRP cutoff value in predicting coronary artery disease in Japanese individuals is 1.0 mg/L^37^. These findings suggest that inflammation present in the PISA ≥ 500 group may sufficiently increase the arteriosclerosis risk. However, the hs-CRP cutoff value for predicting coronary artery disease in Caucasians is higher than that in Japanese. Moreover, the average hs-CRP is higher than that of Japanese. Therefore, the effect of hs-CRP due to periodontal tissue inflammation shown in PISA may differ among races, as well as the degree of effect on systemic diseases. Hence, future studies conducted worldwide is warranted.

Periodontitis have a significant correlation with serum CRP^8–13^. And BOP has been associated with hs-CRP^38,39^. In this study as well, the number of BOP-positive sites was found to be significantly related to hs-CRP. However, the degree of inflammation caused by periodontitis could not be measured, hindering medical and dental collaboration. PISA is an index that quantifies the degree of inflammation based on the results of periodontal tissue examination. However, PISA does not have a clear standard for the severity of periodontal disease or a standard for its systemic effects.

A study reported an average PISA of 2309.42 ± 587.69 mm^2^ and 1697.8 ± 749.9 mm^2^ in patients with severe and moderate periodontitis, respectively, which is higher than our average PISA^40^. We found no significant correlation between hs-CRP levels and the number of teeth with PPD ≥ 6 mm. This could be attributed to the participants being recruited as part of a longevity study and the small number of patients with severe periodontitis. According to the subject questionnaire, 150 people (94.9%) had a family dentist, which may be due to the fact that there were more people who were relatively conscious of their dental health than the general population. Additionally, PPD and alveolar bone resorption do not indicate the current inflammatory state of periodontal tissue; therefore, it is difficult to reflect them. However, the significant between-group difference in the hs-CRP levels suggests that the PISA is a more sensitive index that affects hs-CRP.

Additionally, the significant association observed between the PISA and hs-CRP could have resulted from the small number of obese participants. There was no significant difference in the median BMI between the PISA < 500 group (21.9 kg/m^2^) and the PISA ≥ 500 group (22.4 kg/m^2^). Severe obesity causes inflammation in visceral adipose tissue; further, it increases CRP levels. Observational studies in the United States have reported that among participants with a BMI 20 kg/m^2^, in the regression model, those with severe periodontal disease have approximately twice the CRP levels as those in participants with no or mild periodontal disease; however, this difference diminishes with increasing BMI, and inflammation caused by periodontal disease has been reported to be completely masked by the effect of obesity upon the rise of the BMI to approximately 35 kg/m^2^^41^. A Japanese survey on type 2 diabetes reported a positive correlation of antibody titers against periodontal disease bacteria with hs-CRP levels when the BMI was < 27.0 kg/m^2^; however, there was no correlation when BMI exceeded that value^42^. An interventional study in the United States showed negative results for improvement of HbA1c with periodontal treatment; however, the mean BMI of the participants in that study was around 35 kg/m^2^, and they were morbidly obese^43^. Since obesity induces inflammation, the inflammatory response caused by periodontal disease can be completely masked by obesity-related inflammation in morbidly obese patients^41,42,44^. These findings indicate that the effects of periodontal disease have spread to adipose tissue and that periodontal inflammation is amplified in the portal vein area.

In this study group, there were 27 subjects diagnosed with diabetes. However there was no significant difference in hs-CRP between diabetic and non-diabetic subjects. Diabetes was controlled to some extent by medication and diet, and in one subject, it was uncontrolled with HbA1c 8.0 or higher. Therefore, we believe that there was no association between diabetic patients and hs-CRP.

Thirty-four subjects were taking statins, 5 were taking NSAIDs, and 5 were taking antibacterials, and 42 subjects were taking any of these medications. We compared hs-CRP between the 42 subjects taking the drugs and those not taking the drugs and noted no significant difference. Although there may be a decrease in CRP due to medication in each individual, we believe that the decrease in hs-CRP due to medication was not enough to cancel out the correlation between PISA and hs-CRP.

It has been reported that PISA is positively correlated with hypertension^17^. Blood pressure data was not available in all the subjects of this study. Analysis showed that hypertensive subjects tended to have higher PISA than non-hypertensive subjects.

Regarding chronic kidney disease and periodontal treatment, an observational study on hemodialysis patients reported that periodontal treatment significantly improved blood CRP levels^45^. Moreover, among patients with periodontitis with comorbid end-stage renal disease, the hs-CRP levels of patients with PISA of ≥ 490.56 mm^2^ were 3.26 times more likely to exceed 5 mg/L^18^. Additionally, it has been reported that saliva metabolite is significantly increased at PISA of ≥ 550 mm^2^^46^. Regarding rheumatoid arthritis (RA), patients with PISA of > 550 mm^2^ had significantly higher odds ratios for severe RA compared to patients with PISA of ≤ 550 mm^2^^47^. As described above, several papers have reported that periodontal tissue inflammation can affect disease and systemic markers near PISA of 500 mm^2^.

In the present study, a significant difference in hs-CRP was observed between the two groups based on PISA of 500 mm^2^, and a significant difference in hs-CRP was observed between the two groups based on PISA of 600 mm^2^. However, when the reference value was lowered to PISA of 400 mm^2^, the difference between the two groups decreased and no significant difference was noted in the hs-CRP between the two groups.

Therefore PISA = 500 mm^2^ could be the limit value that affects systemic inflammatory markers. With further research, we may be able to predict to some extent the CRP of patients with PISA ≥ 500 without blood sampling. And this results may help to elucidate the causes of various chronic inflammatory diseases. Furthermore, it may be easier for dentists and internists to work together by using PISA as a common language and sharing the risk of systemic disease from periodontitis. In addition, PISA, along with BMI, which is an indicator of obesity, may be a simple, easy-to-use marker for mild inflammation throughout the body. Moreover, for Japanese who tend to have low BMI and hs-CRP^33,48–51^, PISA may be a particularly useful marker of hs-CRP.

If further research is carried out, and if PISA is recognized as a useful marker not only for periodontal disease but also for hs-CRP, it will be a bridge from dentistry to medicine and will have great social significance.

However, this study had several limitations. First, it was conducted as part of a study on healthy longevity, and few participants had periodontitis with high PISA. Compared with that of the PISA < 500 group, the PISA ≥ 500 group showed significantly higher hs-CRP levels; however, more detailed data could have been obtained if more participants with high PISA values were enrolled. Additionally, this was an epidemiological study targeting limited areas, fixed ages, and Japanese people. There is a need for future related studies using wide geographical areas and age ranges. Future longitudinal studies may allow a more detailed investigation of the association between periodontal tissue inflammation and systemic diseases.

Our findings further confirmed the association between the PISA and hs-CRP levels. Moreover, this marker could be more sensitive than the severity observed on conventional periodontal examinations. This could serve as an index for evaluating the degree of influence of periodontitis on the whole body, enabling medical and dental cooperation.
